# Myiasis absent *Wohlfahrtiimonas chitiniclastica* bacteremia in a lung cancer patient: a case report

**DOI:** 10.1186/s40001-021-00576-w

**Published:** 2021-09-08

**Authors:** Peter Dovjak, Michael Kroißenbrunner, Bernhard Iglseder

**Affiliations:** 1 Department of Geriatric Medicine, Salzkammergut Clinic Gmunden, Miller von Aichholzstraße 49, 4810 Gmunden, Austria; 2 Institute of Pathology, Salzkammergut Clinic Vöcklabruck, Dr.-Wilhelm-Bock-Straße 1, 4840 Vöcklabruck, Austria; 3 Department of Geriatric Medicine, Christian-Doppler-Clinic, Paracelsus Medical University, Ignaz-Harrer-Straße 79, A-5020 Salzburg, Austria

**Keywords:** *Wohlfahrtiimonas chitiniclastica*, Malnutrition, Myiasis, Musca domestica, *Wohlfahrtiimonas chitiniclastica*, Malnutrition, Myiasis, *Muscadomestica*

## Abstract

**Background:**

A gruesome infection was found in a woman with advanced lung tumor and associated malnutrition. Worldwide, bacteremia with *Wohlfahrtiimonas chitiniclastica* was only found in 13 cases yet.

**Case presentation:**

This is the first case in Austria and the first case without infestation of maggots.

**Conclusions:**

This germ is an emerging human pathogen not only in patients with poor personal hygiene, difficult social circumstances, alcohol dependence or chronic wounds. It must be included in the differential diagnosis of immunocompromised patients with pneumonia.

## Abstrakt

**Hintergrund:**

Eine grauenhafte infektion wurde bei einer patientin mit fortgeschrittenem lungentumor und malnutrition gefunden. Bisher wurden weltweit erst 13 fälle mit einer *Wohlfahrtiimonas chitiniclastica*—bakteriämie beschrieben.

**Fallbericht:**

Dieser erste Fall in Österreich manifestierte sich ohne eine infestation mit maden.

**Konklusion:**

Dieses bakterium ist ein neuer krankheitserreger der nicht nur bei patienten unter mangelnder hygienebedingungen, schwierigen sozialen verhältnissen, alkoholabhängigkeit oder chronischen wunden auftreten kann. Es muss in die differentialdiagnostik immunkompromittierter patienten mit pneumonie miteinbezogen werden.

## Background

The new gammoproteobacteria *Wohlfahrtiimonas chitiniclastica* was isolated in 2008 by Tòth et al. from a fly larvae of the parasitic fly *Wohlfahrtia magnifica* [1]. This germ is a Gram-negative, regular, non-motile, straight, short rod with strictly aerobic growth. The parasitic fly lives in vertebrates and was first isolated in Eastern Europe, Mediterranean area, and Middle East. Further vectors include *Chrysomya megacephalea, Lucilia sericata and Musca domestica*. The name of the germ refers to the strong chitinase activity described by Tòth et al. [1]. The germ is used as biomarker in forensic cases [2]. In human 13 cases of human infection have been published yet including the first case of *Wohlfahrtiimonas chitiniclastica* bacteremia in continental United States derived from infection of open chronic wound associated with poor care and hygiene [3, 4].

## Case presentation

A 79-year-old female presented to the department of acute geriatric medicine due to unintended weight loss of 6 kg in the last 6 months, loss of appetite and fatigue. The medical history was remarkable: exactly 2 years before admission she was admitted to the department of internal medicine in the same hospital because of thoracic pain on the right side with an intensity of 10 in the visual analog scale. In the computed tomography a spiculated pulmonary lesion of 30 mm diameter was detected in the right anterolateral upper lobe of the lung. The radiologist classified the lesion highly suspicious for cancer. A positron emission tomography confirmed the suspicion for cancer due to high activity in the fluorodeoxyglucose metabolism. A newly detected pleural effusion was drained and amber fluid with bloody spots was analyzed. It revealed a mass of neutrophilic granulocytes, histiocytes and mesothelial cells, findings that were classified as reactive for inflammation. No tumor cells were found. Cultures for the detection of tuberculosis and anaerobic or aerobic germs were negative. Aminopenicillin was given parenterally and an appointment was made for bioptic clarification of the lesion for outpatients, an appointment that was not perceived because of negative attitudes of the patient against an invasive procedure. Therefore, no exact diagnosis of the tumor could be made. No further decision making process between physician and patient`s relatives were documented.

Two years later, on admission to the department of acute geriatric medicine, she had a reduced general condition. On her right upper field, the respiratory sound was attenuated. Her skin was pale, and she was emaciated. The body mass index was 16 (ideal value 24–29, age and sex adjusted).

In the comprehensive geriatric assessment, we found 18.5 score points in the Mini Nutritional Assessment (range 0–30 points, lower points indicating malnutrition), indicating malnutrition and 95 score points (range 0–100 points, higher points indicating higher performance status) in the Barthel index, indicating the self-care uncompromised. The cognitive tests (Mini Mental test and Clock Performance Test) were unremarkable.

A repeated thoracic computer tomography indicated an enlargement of the lesion in the right upper lobe of the lung to 9 × 9 × 7.5 cm diameter with a similar lesion in the right lower field of the lung being highly suspicious for metastasis. The lesion was spiculated, with an unshaped border and lobulated. The draining bronchus stopped abruptly in front of the lesion. The lymph nodes were normal; we noted no destruction of the adjacent ribs.

Laboratory findings of the peripheral venous blood sample were as following: leucocytes 11,300/mm^3^ (86.6% neutrophils); hemoglobin count 9.9 g/dl; platelet count 3,34,000/mm^3^.

Her serum chemistry results were as follows: [Na^+^], 135 mmol/l; [K^+^], 4.3 mmol/l; estimated glomerular filtration rate (MDRD formula) 51 ml/minute; C-reactive protein 115 mg/l. The results of liver metabolism were unremarkable. The test for COVID-19 virus (polymerase chain reaction test) was negative as well as the test for *Mycobacterium tuberculosis* (QuantiFERON^®^-TB Gold Test).

After drawing samples for blood cultures, a calculated therapy for suspected popped up pneumonia was started with ampicillin plus sulbactam parenterally due to the diagnostic workup.

**Fig. 1:**
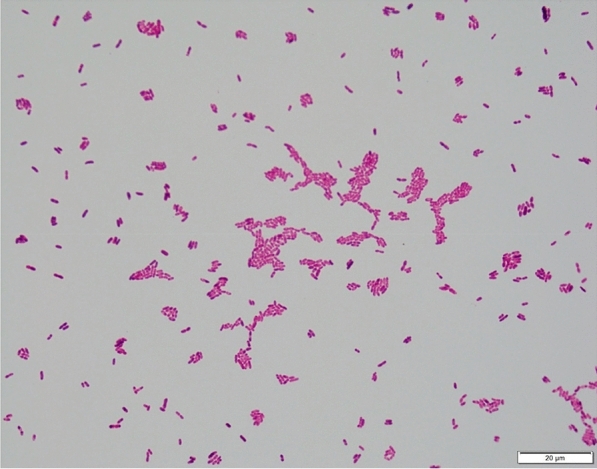
Gram staining: Gram negative rods identified as *Wohlfahrtiimonas* *chitiniclastica* from Columbia Blood Agar 1000 ×

**Fig. 2:**
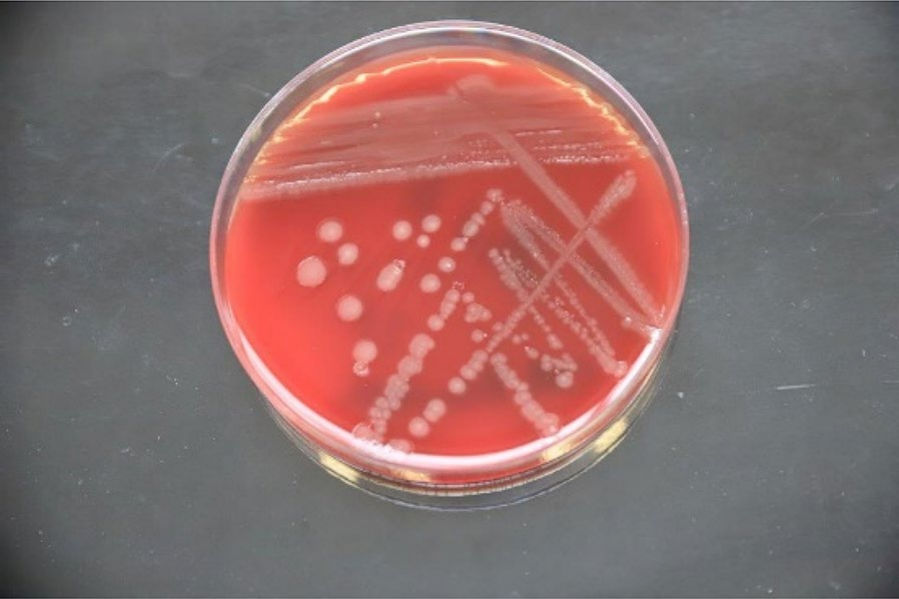
Growth of *Wohlfahrtiimonas* *chitiniclastica* on Columbia Blood Agar

One set of blood cultures were sent to our microbiological laboratory and incubated in the Beckton and Dickinson (BD) Bactec™ Instrument, which provided a positive result for the aerobic bottle after an incubation period of 15 h and 30 min. A Gram staining performed with the positive blood culture and the specimen was plated and incubated accordingly to the manufacturer’s protocol. The Gram staining showed short Gram-negative rods (Fig. 1). After 24 h of incubation at 35 °C under aerobic conditions growth could be seen on PolyViteX Agar, Columbia Blood Agar and MacConckey media (BD) (Fig. 2), showing a mucoid morphology as previously described by Chavez et al. [4]. Cultivated bacteria were further analyzed with a Bruker Microflex MALDI-TOF (Matrix assisted laser desorption ionization–time of flight) mass spectrometer setup. Data which were obtained from the Bruker Microflex setup showed a strong result for *Wohlfahrtiimonas chitiniclastica* according to the score of 2.56 and a consistency rating of A +++  due to the manufacturer`s protocol, using the research version 4.1 model-based testing compass database from Bruker Daltonics. Since the MALDI TOF analysis provided a solid result of  >  2, which secures identification at species level. Therefore, no further diagnostic procedures were necessary to provide the clinician with a feasible result.

To provide guidance for adequate therapy in this case, our laboratory conducted an elipsometer-tests (BD) and interpreted the results according to EUCAST (European Committee on antimicrobial susceptibility testing) PK/PD (pharmacokinetic/pharmacodynamic) non-species related breakpoints (Table 1), since species specific breakpoints were absent [5, 6]. The strain of *Wohlfahrtiimonas chitiniclastica* showed very low minimum inhibitory concentrations for all antibiotics indicating antibiotic treatment could be taken into account.

**Table 1:** Minimum inhibitory concentrations (MIC) testing of *Wohlfahrtiimonas* *chitiniclastica* for selected substances

Antibiotic	MIC (µg/ml)	Remark for clinical use
Amoxicillin/clavulanic acid	0.12	Yes
Piperacillin/tazobactam	0.19	Yes
Cefotaxim	0.012	Yes
Cefepime	0.023	Yes
Imipenem	0.19	Yes
Ciprofloxacin	0.016	Yes
Trimethoprim/sulfmethoxazole	0.19	Not applicable

Along with the blood culture results an additional exposure history was performed. The results showed that the patient lived with a dog and was a cigarette smoker with an exposure of 30 pack years and denied drug use. The patient remained afebrile and without pain. Her chief complaints were fatigue and exhaustion. Blood cell counts and parameters of inflammation remained unchanged despite the advanced oncologic situation. We initiated nutritional support using supplements and a diet adjusted to the recommendations of the dietologist. Detailed consultation on further diagnostic and therapeutic options followed on several occasions and clinical visits were accompanied by the patient’s cohabitant. For personal reasons, the patient refused further diagnostic or therapeutic measures. In accordance with the patient’s wishes, we constituted a palliative care and finalised the antibiotic course after 8 days without consecutive laboratory controls. The further clinical course after discharge from hospital was unremarkable according to the report of her family doctor. Due to the increased need of care, the patient`s cohabitant established home nursing. The patient died 19 days after the discharge from hospital; no autopsy was performed due to the order of the family.

## Conclusions and discussion

Here we present the first case of a *Wohlfahrtiimonas chitiniclastica* bacteraemia in Austria in a terminally ill cancer patient. To our best knowledge, this is the first case of infection with *Wohlfahrtiimonas chitiniclastica* without an infestation with maggots. Myiasis typically involves skin and soft tissues, either primarily or in wounded skin. It occurs in patients with multimorbidity, mental retardation, alcoholism, diabetes mellitus, basal cell carcinoma, and vascular occlusive disease [7]. Maggots has also been used for centuries to promote debridement and healing of wounds as a therapeutic approach [8]. Notably our patient showed no history of having suffered from myiasis or a therapy with maggots. The majority of recent published cases reported myiasis in patients who have lived under poor hygienic conditions and suffered from open skin wounds [9]. In contrast our case patient had an intact integument. Unfortunately, no obvious infection pathway was identifiable, since the patient lacked myiasis. Nevertheless, several meaningful observations can be offered in our case:

In our opinion, the most potent risk factor in this case was malnutrition, which is a risk factor for poorer outcome among patients with infections. A retrospective observational cohort study extracted from a national database analysed 3,34,266 patients with sepsis, showing that the odds were increased up to 297% [10]. Efforts, involving all health care providers in the prevention, identification, and treatment of malnutrition in community-dwelling people before hospitalization might mitigate against these devastating outcomes, but in this case, malnutrition was associated with advanced and progressing lung tumour with high suspicion for lung cancer. To our regret, we could not persuade our patient for further diagnostic workup and therapy.

One possible source of infection with *Wohlfahrtiimonas chitiniclastica* may be the common housefly, *Musca domestica* (Linnaeus, 1758). It is the most common and widespread fly species in the world. *Musca domestica* lives in close association with humans and is able to complete its entire lifecycle within habitations of humans and domestic animals. The patient in our case lived with a dog. Transmission of pathogens from houseflies to human are well known, which may also cause serious and even life-threatening infections. At least 130 different bacteria, viruses, fungi, and parasites were identified in association with the insect to date [11, 12]. Although, Khamesipour et al. (2018) did not mention the particular bacteria, which had been found in our case in his review article on human pathogens carried by the housefly, *Wohlfahrtiimonas chitiniclastica* was found in myiasis induced by *Musca domestica* and induced a case of fulminant sepsis described by Almuzara et al. (2011) and Parwani et al. (2014) [13, 14]. A similar infection pathway is assumable for our patient. Bacteraemia with *Wohlfahrtiimonas chitiniclastica* in our case was detected in October, the month with the highest activity and spread of *Musca domestica* due to the biology of the insect in Austria [15]. This fact strengthens this assumable infection pathway.

In summery *Wohlfartiimonas chitiniclastica* has to be considered as an emerging human pathogen in patients in a terminal oncologic situation with malnutrition. Only 13 documented cases of human infections have been published in the last decade. This case report represents the first incidence in Austria [3]. The so far published cases of infected patients were all quite impressive: the patients had poor personal hygiene, difficult social circumstances, alcohol dependence or chronic wounds. The sites of isolation have ranged from the skin, bone, soft tissue and blood, but not so in our case. Our patient was a graduated retired business owner of an antique shop, contradicting the previously described living conditions.

## Data Availability

All clinical and microbiological data in this case are electronically stored in the hospital information system.
